# Novel objective-subjective pain assessment score results in decreased opioid prescription after elective spine surgery: a prospective pilot study

**DOI:** 10.1007/s00701-026-06794-7

**Published:** 2026-02-16

**Authors:** Dia R. Halalmeh, Yusuf-Zain Ansari, Arwa Jader, Ashra Mirza, Hazem Eltahawy

**Affiliations:** 1 Department of Neurosurgery, University of Iowa Hospital, Iowa City, IA USA; 2 College of Science and Technology, Temple University, 1801 N Broad St, Philadelphia, PA 19122 USA; 3 Department of Neurosurgery, University of Kufa, Kufa, Iraq; 4 Enhance Center for Interventional Spine and Sports, Livonia, MI USA; 5 Department of Neurosurgery, Central Michigan University Medical School, Saginaw, MI USA

**Keywords:** Pain Scale, Opioid Analgesics, Spine Surgery

## Abstract

**Background:**

Opioid dependence after spine surgery is a major contributor to the opioid epidemic in the United States. Current pain assessment tools are largely subjective, linking patient satisfaction to reported pain and encouraging liberal opioid prescribing. Incorporating objective criteria into pain evaluation may improve prescribing practices while maintaining patient satisfaction.

**Methods:**

The OBSUB scale, a 10-point tool combining subjective pain scores (points 1–5) with objective signs (points 6–9: autonomic activation, avoidance behaviors, postural guarding, and distraction) plus a point for pain exaggeration, was implemented in 44 patients over 3 months. Postoperative analgesics were prescribed progressively according to the WHO analgesic ladder, with emphasis on non-opioid alternatives and staff education. Mean daily Morphine Milligram Equivalents (MME) were recorded for four intervals: 30 days preoperatively, and 0–30, 31–60, and 61–90 days postoperatively.

**Results:**

Compared to institutional pre-intervention averages, mean daily MME decreased by 53.2% in the first postoperative month (P = .30), 32.2% in the second month (P = .0040), and 91.8% in the third month (P = .0085). At 90 days, 76% of patients were no longer prescribed opioids compared to 48% prior to intervention. Despite more restrictive prescribing, patient satisfaction increased by 27% based on Hospital Consumer Assessment of Healthcare Providers and Systems (HCAPHS) scores.

**Conclusions:**

The OBSUB scale, integrating objective with subjective pain measures, effectively reduced postoperative opioid prescriptions without compromising patient satisfaction. This approach offers a promising strategy to mitigate opioid dependence after spine surgery and warrants validation in larger multicenter trials.

## Introduction

In 2016, opioid-related deaths in the United States exceeded 42,000 [20]. The rise of spine surgeries has brought with it complications such as opioid dependence, exacerbating the opioid abuse epidemic in the United States [10]. Many patients are opioid-dependent before spine surgery due to prolonged conservative pain management, often mandated by insurance companies despite questionable efficacy [11]. Prolonged opioid use before surgery increases the risk of dependence and predicts poor outcomes, long-term reliance, and a higher likelihood of illicit drug use, despite limited evidence supporting their effectiveness for chronic back pain [4, 21–23]. This pre-and postoperative liberal opioid prescribing is amenable to quality-of-care improvement processes.

Current postoperative pain management in spine surgery commonly relies on subjective pain reports, with opioid prescriptions often guided primarily by patient-reported numeric pain scores rather than objective clinical indicators [7]. Standard practice in many institutions includes prescribing short-acting opioids at discharge, frequently in quantities exceeding what is required for acute postoperative recovery. Studies have shown that 30–60% of patients receive long-term opioid routine spine procedures, despite wide variation in actual patient consumption and minimal evidence supporting such liberal prescribing patterns [2]. This reliance on subjective pain scales, combined with institutional pressures to maintain high patient satisfaction scores, contributes to persistent opioid use, overprescription, and the risk of dependence.

Therefore, a more accurate tool to objectify the patient’s pain and subsequently tailor opioid analgesics is necessary to prevent and decrease opioid dependence before and after elective spine surgery. This study aims to examine the role of an Objective-Subjective (OBSUB) Pain Assessment tool in lowering persistent use of narcotic pain medications three months post-surgery and decrease pre- and postoperative opioid use while maintaining patient satisfaction and pain control.

## Methods

### Patients and data collection

Eligibility included all consecutive adult patients who underwent elective or semi-elective spine surgery by the senior author at [BLINDED FOR REVIEW] during the defined six-month time frame. The non-interventional group consisted of consecutive spine surgery patients who underwent surgery during the three months before implementation of the OBSUB pain-assessment protocol, while the interventional group included all consecutive patients who underwent spine surgery during the three months after the OBSUB protocol was put into practice.

Patients were not excluded on the basis of inability to taper or need for unanticipated dose escalation. However, two extreme outliers were excluded from the analysis, one from each group, because they were maintained on unusually high preoperative MME levels that were not representative of the broader and more homogeneous study population. These cases were carefully reviewed, and the decision to exclude them was made to prevent distortion of group averages and to preserve internal validity. All remaining patients were included in the final dataset.

Baseline opioid prescription data were retrospectively collected from 44 patients and were followed up over a period of 4 months. The primary variables collected for analysis included pain scores, opioid consumption, patient satisfaction metrics, and demographic characteristics. Mean daily Morphine Milligram Equivalents (MME) were obtained from the Michigan Automated Prescription System (MAPS) database and recorded for four intervals: 30 days preoperatively, and 0–30, 31–60, and 61–90 days postoperatively. Changes in MME were calculated as percentages. Persistent narcotics use after 90 days was analyzed as a Yes/No variable using contingency analysis. Patient satisfaction was assessed using normalized Hospital Consumer Assessment of Healthcare Providers and Systems (HCAPHS) scores (0–100). Statistical significance (P < 0.05) was evaluated with paired t-tests using IBM SPSS Statistics, version 29.0.

### Interventions

**Fig. 1:**
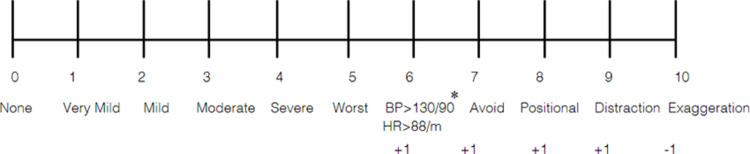
Objective-Subjective (OBSUB) scale for pain assessment in spine patients. The last 5 points are purely objective and can be measured easily with mild or absent interrater variation. * In normotensive patients. For patients with hypertension or unknown baseline BP, an increase > 5–8% from baseline satisfies this criterion

The OBSUB scale was formulated through multiple structured clinical sessions involving iterative review of its components and their real-world applicability. A new 10-point pain scale that includes objective pain assessment in addition to the patient’s subjective score was developed as shown in Fig. 1. The first five points correspond to the patient’s subjective pain experience, modeled after the visual analog scale (VAS). The remaining four points incorporate objective clinical signs of pain. The objective elements (points 6 to 9) are as follows:

- 6. Signs of sympathetic nervous system activation including a sudden increase in blood pressure (BP) (SBP > 130 mmHg and/or DBP > 90 mmHg in normotensive patients, or a rise of ≥ 5–8% from baseline in those with hypertension or unknown baseline BP) and tachycardia (HR > 88/min) [19]. These thresholds were based on standard definitions of hypertension, which define elevated blood pressure as systolic BP > 130 mmHg or diastolic BP > 80–90 mmHg [25]. Changes due to factors like anxiety, medication effects, stress, or other conditions were excluded, as they do not indicate sympathetic activation. Accurate assessment requires reviewing the patient’s medical history, baseline measurements, and potential confounders to interpret BP and HR changes reliably.
- 7. Avoidance behaviors to minimize the pain (eg, low soft voice, slow and gradual movement due to fear of movement-induced pain). Other signs include en bloc turning (head, trunk, and extremities all turn together), shallow breathing, wearing no clothes over the painful area, and inability to eat or drink. These signs reflect subconscious attempts to prevent exacerbation of pain and are consistent with findings in postoperative behavioral pain assessment studies [13]. They can be reliably identified by trained observers with minimal interrater variability, making them practical and reproducible markers of clinically significant discomfort.
- 8. Positional or postural preference to guard the painful region. This is typically manifested as asymmetrical postures or gait disturbances (eg, wide-based gait, limping, reduced arm swing). Other features include sitting on the edge of a chair, and leaning forward while sitting or standing. These behaviors correlate with mechanical pain patterns and are commonly observed in musculoskeletal and spinal pain syndromes. When assessed in context with the patient’s known pathology, these signs offer objective support for the presence and localization of pain.
- 9. Pain distraction by turning the focus of the brain away from the source of the pain to certain activities such as clenching, repetitive movements, fidgeting, rubbing the affected area, and skin mottling due to excessive use of heat pads. These behaviors are adaptive coping mechanisms and align with clinical literature on non-verbal pain communication [17]. They are visually apparent, consistent across patient populations, and easy to document, especially in the perioperative setting where provider-patient interaction is continuous.
- 10. The final point on the scale allows the examining clinician to account for exaggerated pain responses. This point may be subtracted from the total score if such exaggeration is clearly evident. In cases without exaggeration, a score of 0 or 1 may be assigned at the provider’s discretion.

**Fig. 2:**
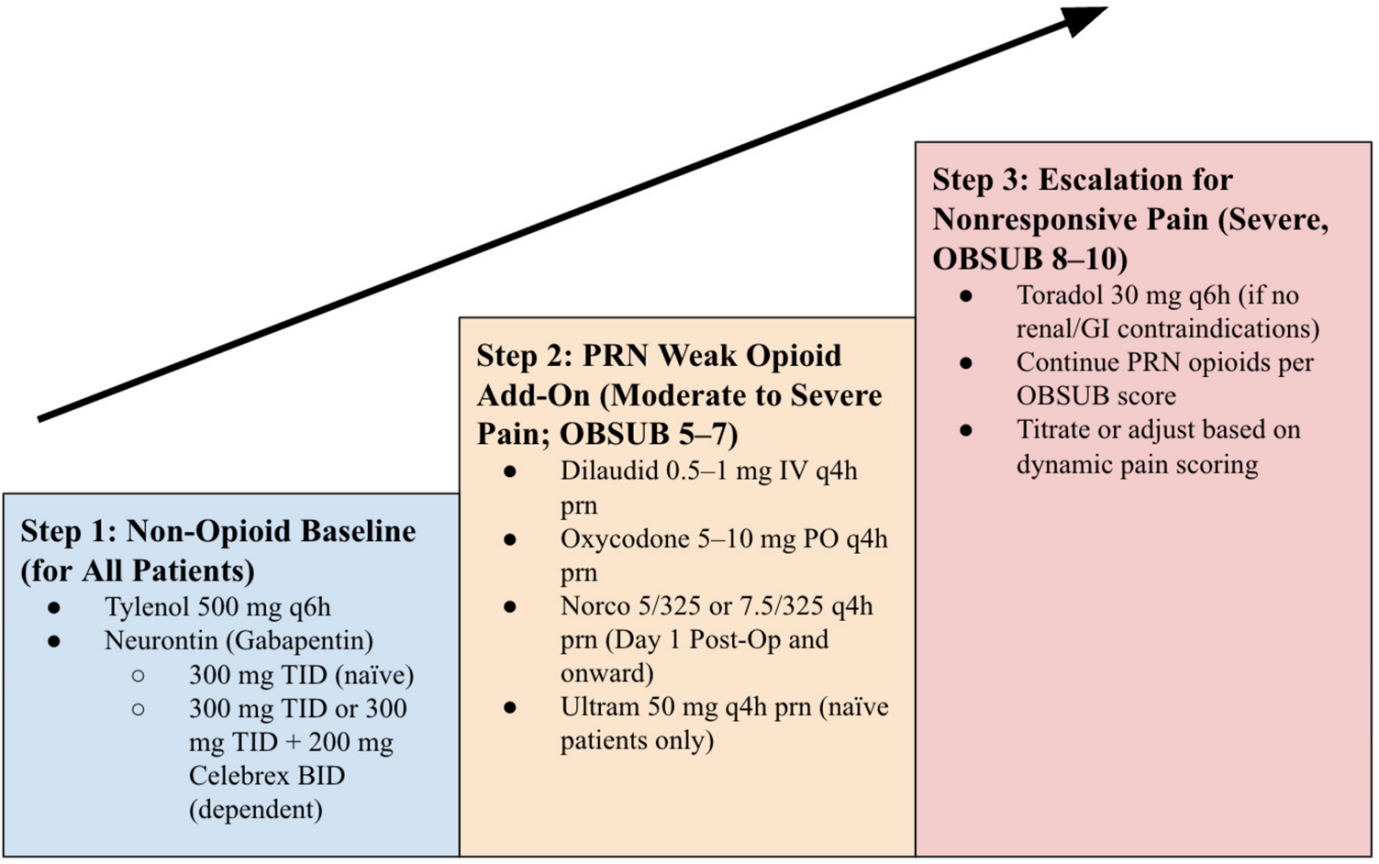
OBSUB-Guided Non-Opioid and PRN Opioid Pain Management Ladder

Postoperative analgesics were prescribed progressively according to the WHO analgesic ladder modified using OBSUB protocol in Fig. 2 [1]. Patients with OBSUB scores of 0–4 received non-opioid analgesics, such as paracetamol or NSAIDs, with or without muscle relaxants. For scores of 5–7, weak opioids like hydrocodone/acetaminophen (up to 7.5 mg) or codeine/acetaminophen were added, while scores of 8–10 warranted stronger opioids such as oxycodone or morphine, with doses adjusted to pain patterns. The dose and schedule of these short-acting opioids were adjusted to the patient’s pain pattern. In addition, a maximum of 45 pills of opioids were prescribed upon discharge for the first month with refills tapered to 30 pills and 15 pills for the subsequent 2 months and eventually stopped after 3 months. Non-opioid strategies included local anesthetic wound pumps, TENS, ultrasound, massage therapy, and lidocaine patches.

**Fig. 3:**
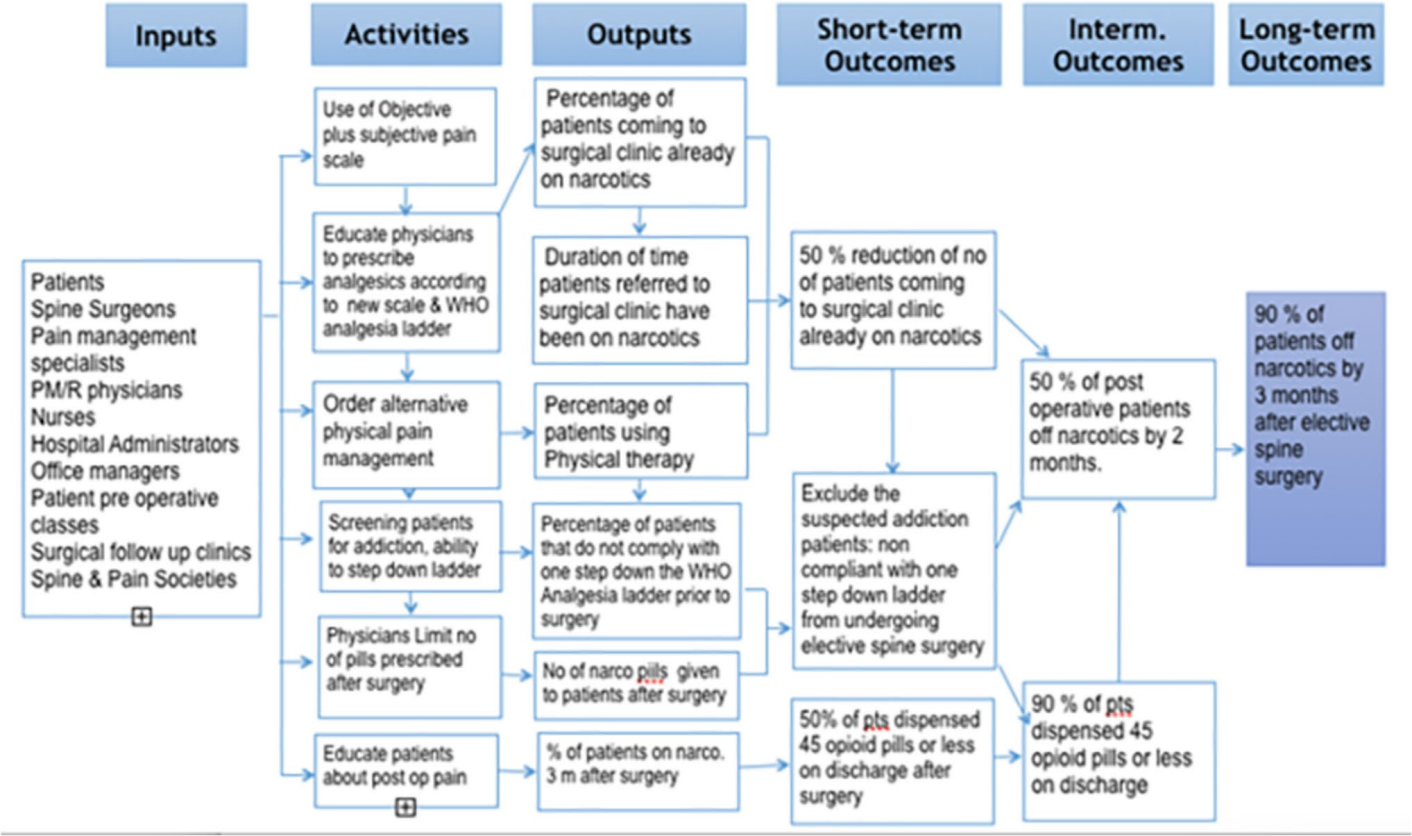
Illustration showing the interventions performed, including application of OBSUB scale, to limit postoperative opioid prescription after elective spine surgery

Implementation involved patients, families, healthcare providers, administrators, and nurses who were trained to record pain scores. Adherence to the WHO ladder was essential to minimize opioid prescriptions and dependence. Primary care physicians were educated on refraining from narcotic prescriptions pre-intervention, setting expectations, and promoting non-opioid alternatives. Figure 3 summarizes the interventional steps used to enhance opioid management and maintain adequate pain control. The evaluation of pain severity was conducted using the newly developed OBSUB scale. When discrepancies arose between the prescribed narcotic dose or potency and the patient’s reported pain, treatment was adjusted through de-escalation along the WHO analgesic ladder. Patients who were unable to comply with this protocol were not considered surgical candidates and instead underwent additional assessment for possible substance misuse. In such cases, conservative management of their spinal condition was continued.

## Results

Forty-five consecutive patients (24 female, 21 male) who underwent elective or semi-elective spine surgery in a single institute were evaluated prospectively. The characteristics of patients included in the study are summarized in Table 1. The mean age of the study population was 64.8 ± 13.6 (range 26—95 years), and more than half (54.5%) of treated patients were women. A total of 74 levels were treated, and the two most frequent levels were L4-L5 (25.6%, n = 19) and L5-S1 (12.1%, n = 9), making the lumbar region the most common treated spine region, followed by cervical and thoracic spine. The most common surgical intervention was laminectomy (54.5%, n = 24) followed by hemilaminectomy (9.0%, n = 4), screw removal/revision (4.5%, n = 2), corpectomy (6.8%, n = 3), arthroplasty (2.3%, n = 1), and foraminotomy (2.3%, n = 1); with or without instrumented stabilization/fusion (63.6%, n = 28). The overwhelming majority of these procedures were performed posteriorly (81.8%, n = 36) whereas anterior approach (anterior cervical discectomy and fusion (ACDF) or anterior lumbar interbody fusion (ALIF)) was performed in 18.2% of patients (n = 8). The mean OBSUB score decreased by approximately 25% at the first follow-up visit following surgery.

**Table 1:** Demographics and clinical characteristics

Characteristic	All patients (n = 45)	Non-interventional group (n = 30)	Interventional group (n = 15)	P-value
Age (years)				
Mean ± SD	64.8 ± 13.6	65.7 ± 12.9	65.0 ± 14.1	0.86
Range	26—95	32–95	27–81	-
Gender, number (%)				
Female	24 (53.3%)	16 (53.3%)	8 (53.3%)	NS
Male	21 (46.7%)	14 (46.6%)	7 (46.6%)	NS
Number of Levels Treated, number (%)				
1-level	26 (57.7%)	16 (53.3%)	10 (66.7%)	NS
2-level	10 (22.2%)	7 (23.3%)	3 (20.0%)	NS
3-level	5 (11.1%)	5 (16.6%)	0	0.11
4-level	3 (6.7%)	2 (6.7%)	1 (6.7%)	NS
5-level	1 (2.2%)	0	1 (6.7%)	NS
Spinal Region Treated, number of levels (%)	75	53	25	
Lumbar	43 (57.3%)	33 (62.2%)	9 (36.0%)	0.13
Cervical	30 (40.0%)	17 (32.0%)	13 (52.0%)	0.19
Thoracic	5 (6.7%)	2 (3.8%)	3 (12.0%)	0.23
Sacral	18 (24.0%)	15 (28.3%)	4 (16.0%)	0.31
Surgical Intervention				
Laminectomy	25 (55.5%)	16 (53.3%)	9 (60.0%)	NS
Hemilaminectomy	4 (8.9%)	2 (6.7%)	2 (13.3%)	NS
Screw removal/revision	2 (4.4%)	2 (6.7%)	0	NS
Corpectomy	3 (6.7%)	3 (10.0%)	0	0.22
Arthroplasty	1 (2.2%)	1 (3.3%)	0	NS
Foraminotomy	1 (2.2%)	1 (3.3%)	0	NS
Stabilization/fusion**	21 (46.7%)	15 (50.0%)	6 (40.0%)	NS
Surgical Approach, number (%)				
Posterior	36 (80.0%)	23 (76.7%)	13 (86.7%)	NS
Anterior	9 (20.0%)	7 (23.3%)	2 (13.3%)	0.52
Average daily MME over 1 month prior to intervention/surgery	35.5	42.08	22.9	0.30

* SD: standard deviation; MME: morphine milligram equivalents

** Stabilization/fusion procedures included anterior cervical discectomy and fusion (ACDF); anterior lumbar interbody fusion (ALIF); and simple posterior fusion with or without laminectomy

**Fig. 4:**
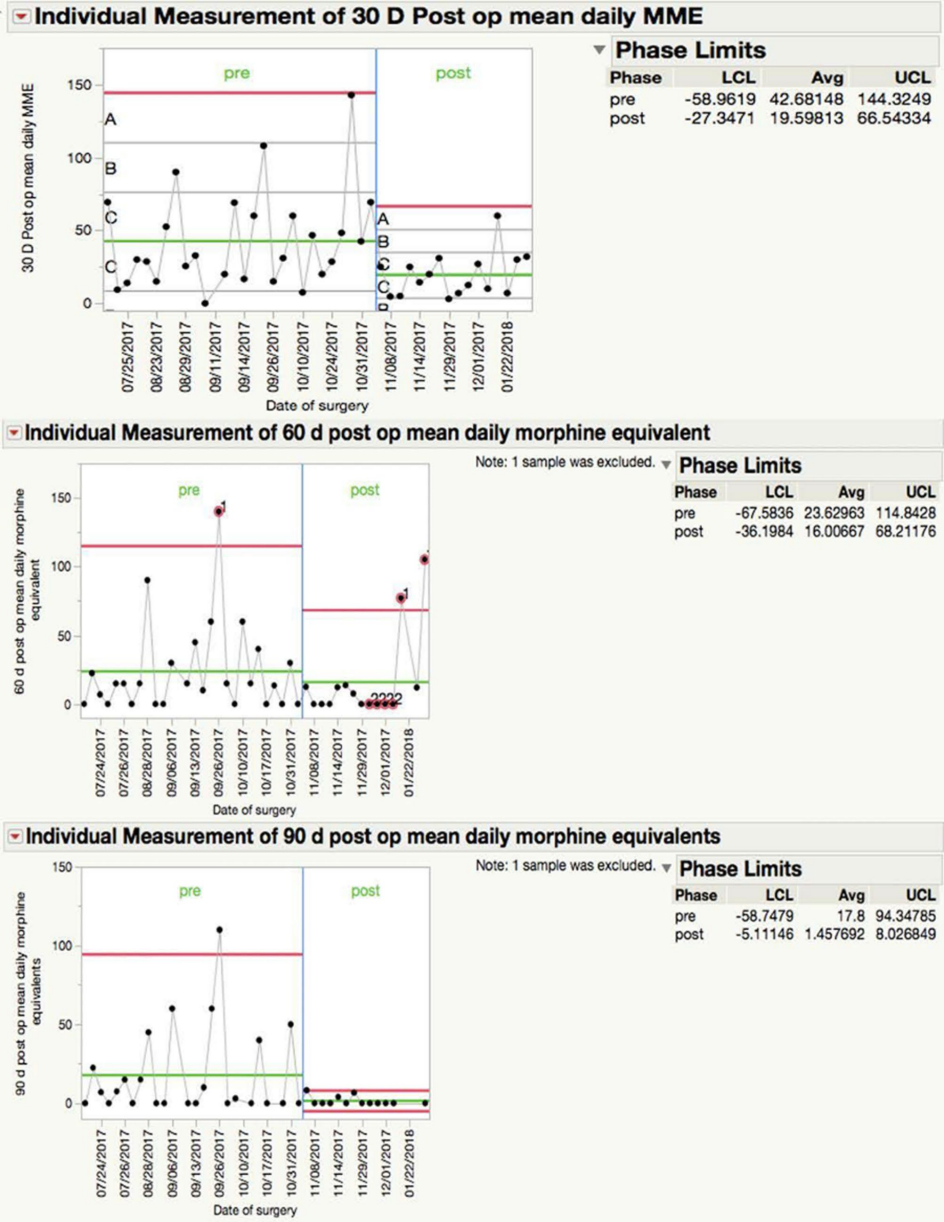
Graph showing snapshots of the three postoperative MME periods as compared to institutional values prior to implementation of the interventions

**Fig. 5:**
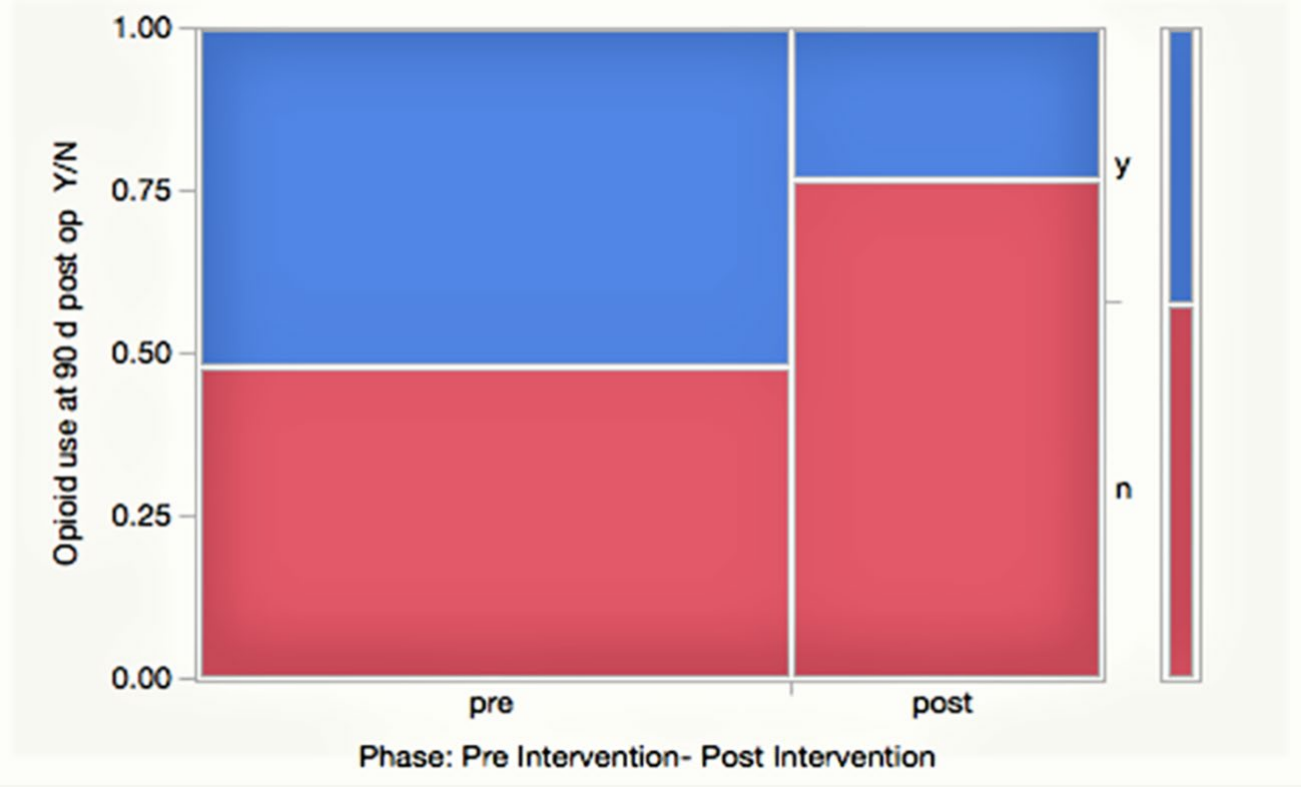
Mosaic plot showing results for contingency analysis of persistent opioid use at 90 days after surgery

The average OBSUB score for all patients prior to surgery was 5.9 ± 2.9. Table 2 summarizes the percentage reduction in mean daily MME following implementation of the interventions. Compared to the average institutional MME prior to intervention (Fig. 4), the mean daily MME for the study population was reduced by 53.2% during the first postoperative month, 32.2% in the second postoperative month, and 91.8% in the third postoperative month. Based on the HCAPHS scoring system, patient satisfaction was similar to or better than that prior to interventions for each interval (Table 2). The mean normalized, 3-month HCAPHS score was 76.7, which increased from the pre-interventional mean of 56 (27% increase). There was a 58.3% overall increase in patients not prescribed opioid medications by 90 days postoperatively as demonstrated in the contingency analysis (48% increased to 76%) (Fig. 5).

**Table 2:** Summary of mean daily Morphine Milligram Equivalents (MME) and Hospital Consumer Assessment of Healthcare Providers and Systems (HCAPHS) scores for patients undergoing elective spine surgery in a single institute over the study period (N = 44)

	Before Intervention (n = 30)	After Intervention (n = 15)	Percentage Reduction in Daily MME	*P*-value*	Average HCAPHS score Before Intervention**	Average HCAPHS Score After Intervention**
Mean Daily MME during the 30 days preoperatively	42.08	22.9	45.5%	0.30	-	-
Mean Daily MME between day 0–30 postoperatively	42.68	19.95	53.2%	0.30	68	50
Mean Daily MME between day 31–60 postoperatively	23.62	16.00	32.2%	**0.0040**	50	80
Mean Daily MME for between day 61–90 postoperatively	17.8	1.45	91.8%	**0.0085**	50	100

* The p-value was calculated based on a comparison of MME means between pre-and post-interventional groups for each interval

** HCAPHS scores were normalized to a scale of 0–100 based on the patient’s perceived quality of care and satisfaction with 100 being the maximal positive response

## Discussion

Opioid deaths in the United States quadrupled from 1999 to 2015, with prescriptions contributing to half of these deaths [6]. This crisis partly stems from the liberal opioid use in non-cancer patients starting in the 1980 s, driven by concerns over pain control and patient satisfaction [12]. Because adequate pain control is directly related to patient satisfaction and length of stay after surgery, hospital reimbursements for surgeons became heavily influenced by these factors [5]. As a result, the HCAPHS scale for patient satisfaction reflects patient-perceived outcomes which are indirectly linked to pain control due to the subjective nature of pain assessment scales. Unfortunately, the relatively unrestricted prescription of opioids in this patient population has further contributed to the opioid crisis with 225 million prescriptions being dispensed in 2010 alone in the United States [8]. This study sought to address these issues by tailoring opioid management to objectively assessed pain severity through the OBSUB scale, reducing dependence after elective spine surgery.

Given the subjective nature of pain and the serious consequences associated with opioid overprescription, development of an instrument that incorporates the objective components of pain offers an opportunity to standardize pain assessment, reduce interrater variability, and guide more precise analgesic prescription practices. Such an approach also mitigates the risk of over-reliance on subjective reporting, which can be influenced by psychological factors, cultural perceptions, or patient expectations [14, 15]. Integrating these objective measures into clinical workflows has the potential to align pain management with evidence-based guidelines, ensuring that opioid prescriptions are both judicious and tailored to genuine physiological indicators of pain.

Rosenthal et al. used the data of a state-wide prescription monitoring program to identify the risk factors for prolonged opioid use and requirement of higher opioid doses after spine surgery [18]. They found that persistent prescription at 6 months after surgery has been significantly associated with preoperative opioid use [18]. In our study, all patients used opioids preoperatively for pain control (as part of their initial conservative management), however, at 3 months postoperatively, 75% of patients were opioid-free, which increased from 48% prior to intervention. Physiological signs of pain best correlate with acute fluctuations of pain. In chronic pain perception, the sympathetic pain response may adapt with return of vital signs to baseline, but other behavioral or psychological changes may develop to cope with the persistent pain stimuli. This was taken into consideration by including positional changes and avoidance behavior in the OBSUB scale, facilitating its use in the setting of acute and chronic pain changes.

Successful implementation of measures to reduce postoperative opioid prescriptions often requires higher-level legislation. While state-wide policies have shown limited impact beyond the first 30 days post-surgery, our analysis demonstrated significant reductions in opioid use during the second and third months (P = 0.0040, P = 0.0085) [16]. Education of patients and providers, along with the use of the OBSUB scale, was key to ensuring adherence. Maximizing non-opioid medications before considering opioids synergizes with the OBSUB scale, reducing reliance on opioids, lowering dependence, shortening hospital stays, and cutting healthcare costs [9]. A multimodal approach remains essential for effective pain management and mitigating opioid use disorder.

Although opioid prescriptions were restricted during the early postoperative period (e.g., the first 30 days), ensuring adequate pain control remains essential due to its significant impact on hospital stay duration, patient quality of life, and overall outcomes [9, 24]. The analysis showed no statistically significant difference in opioid use during the first 30 days after surgery compared to the 30 days before surgery (P = 0.30). However, a notable 50% reduction in mean daily MME was achieved compared to pre-intervention institutional averages. It is also important to acknowledge that patients in the intervention group had nearly double the preoperative MME use compared to the non-interventional cohort, which may represent a potential confounder when interpreting postoperative reductions. Despite this higher baseline opioid exposure, the intervention group demonstrated substantial declines in MME use over the 90-day postoperative period.

Given that many patients are opioid-dependent before surgery, the role of surgery in improving quality of life and alleviating pain should be carefully evaluated and discussed with patients. Some patients may pursue surgery to address withdrawal symptoms or manage an increased threshold for euphoria. Spine surgery, known for its significant tissue disruption and associated pain, often necessitates high doses of postoperative opioids [18]. This has led to overuse of surgery in patients with refractory pain, which can be challenging to differentiate from pain caused by radiculopathy or neural origin.

Managing expectations for pain relief is essential, particularly in patients with preoperative opioid dependence or chronic pain. While spine surgery is often perceived as a solution for alleviating pain, it is important to emphasize that the primary goals are to improve functionality and quality of life, rather than completely eliminate pain [3]. A study by Canizares et al. found that over 80% of patients undergoing surgery for degenerative spine conditions expected at least some improvement across multiple domains, including physical capacity, independence, and pain reduction. While the majority of patients expected improvements in pain and functionality, a significant portion prioritized overall well-being and independence [3]. Patients should be counseled preoperatively about the potential for residual pain and the need for ongoing pain management, including non-opioid approaches.

### How to improve patient care

Patients in this study may act as a pilot sample for a wider-scale application across the country. Adoption of a more objective pain scale and a tighter controlled opioid analgesic prescription may help curb the prescription opioid epidemic. The new OBSUB pain scale can be used by various providers in outpatient clinics and by nurses in hospitals. Primary care physicians and pain management physicians may be willing to change their established long-term prescription patterns. Surgical clinics should perform patient educational courses to coach patients about post-surgical pain expectation and management. The evaluation of this pain scale should be conducted by spine nurse practitioners and program coordinators. In addition to patients, nurses, and physicians education about the new model, executive decisions by practice owners/administrators and hospital leadership will be needed to implement the new scale.

### Limitations & future directions

This pilot study has several limitations. The small sample size and single-center design limit generalizability and may introduce site-specific bias. Larger multicenter trials are needed to validate findings across diverse populations. Uncontrolled confounders—including demographics, comorbidities, surgical indication and type, prior opioid use, interrater reliability, and psychosocial factors—may have influenced outcomes, underscoring the need for more rigorous screening and adjustments in future studies. The absence of comparisons with other pain assessment tools and randomized groups further restricts interpretation and introduces potential selection bias. Physiological indicators such as blood pressure and heart rate, though valuable, are vulnerable to confounders like anxiety, medications, and cardiovascular conditions. While baseline comparisons and controlled measurement conditions minimized these effects, no single metric can capture pain across all clinical settings.

Future research should employ randomized controlled designs, control for prior opioid use, and incorporate usability testing and clinician training. Efforts should also address barriers to clinician adoption and explore integration of automated systems to streamline use in practice.

## Conclusion

In conclusion, our pilot study demonstrates that the innovative OBSUB Pain Assessment Score can successfully minimize opioid prescriptions following elective spine surgery while continuing to deliver proper pain management. While encouraging, more studies with larger sample size, multi-center trials, and longer follow-up are required to validate these results and improve the tool for wider clinical use. This strategy may help surgery patients control their pain better and become less dependent on opioids.

## Data Availability

No datasets were generated or analysed during the current study.

## Abbreviations

OBSUB: Objective-Subjective
MME: Morphine Milligram Equivalents
MAPS: Michigan Automated Prescription System
HCAPHS: Hospital Consumer Assessment of Healthcare Providers and Systems
VAS: Visual Analog Scale
BP: Blood Pressure
SBP: Systolic Blood Pressure
mmHg: Millimeters of Mercury
DBP: Diastolic Blood Pressure
HR: Heart Rate
WHO: World Health Organization
NSAID: Nonsteroidal Anti-inflammatory Drug
ACDF: Anterior Cervical Discectomy and Fusion
ALIF: Anterior Lumbar Interbody Fusion
